# Loss of Rb1 by epigenetic modification regulates expansion of MDSC in cancer

**DOI:** 10.1186/2051-1426-2-S3-P241

**Published:** 2014-11-06

**Authors:** Je-In Youn, Kyungwha Lee, Hai-Chon Lee, Dmitry I Gabrilovich

**Affiliations:** 1 Wide River Institute of Immunology, Seoul National University, Republic of Korea; 2 The Wistar Institute, Philadelphia, PA, USA

## 

Myeloid-derived suppressor cells (MDSCs) are a heterogeneous group of cells with potent immune suppressive activity in cancer and many other pathologic conditions. MDSCs consist of two major subsets: monocytic MDSCs (M-MDSCs) and polymorphonuclear MDSCs (PMN-MDSCs). Each subset of MDSCs is thought to be developed through the separate differentiation pathways. Here, we demonstrated that in a tumor environment a large proportion of M-MDSCs acquired phenotypic, morphological and functional features of PMN-MDSCs while the normal counterpart of M-MDSCs-Ly6C^hi^Ly6G^- ^inflammatory monocytes (Mon) from tumor-free mice differentiate to macrophages (MΦ) and dendritic cells (DCs). This process was mediated by loss of the retinoblastoma (Rb1) protein in MDSCs. Inhibition of Rb1 expression in PMN-MDSCs correlated with the level of histone acetylation of Rb1 promoter. Treatment of PMN-MDSCs with inhibitors of histone deacetylases (HDAC) resulted in the increase in Rb1 expression. In addition, inhibition of HDAC abrogated differentiation of M-MDSCs to PMN-MDSCs, and restored M-MDSCs differentiation towards DCs and MΦ. These results suggest that down-regulation of Rb1 by epigenetic modification plays a major role in expansion of MDSCs in cancer by regulating PMN-MDSCs differentiation from M-MDSCs. Therefore, inhibition of MDSCs accumulation by treatment of HDAC inhibitors may be considered as a potential therapeutic tool in cancer immunotherapy.

